# Iodine Vapor Staining for Atomic Number Contrast in Backscattered Electron and X-ray Imaging

**DOI:** 10.1002/jemt.22435

**Published:** 2014-09-15

**Authors:** Alan Boyde, Fergus A Mccorkell, Graham K Taylor, Richard J Bomphrey, Michael Doube

**Affiliations:** 1 Dental Physical Sciences, Barts and The London School of Medicine and Dentistry, Queen Mary University of LondonLondon, E1 4NS, United Kingdom; 2 Oxford Animal Flight Group, Department of Zoology, University of OxfordOxford, OX1 3PS, United Kingdom; 3 Structure and Motion Laboratory Department of Comparative Biomedical Sciences, The Royal Veterinary CollegeHatfield, Hertfordshire, AL9 7TA, United Kingdom; 4 Skeletal Biology Department of Comparative Biomedical Sciences, The Royal Veterinary CollegeLondon, NW1 0TU, United Kingdom

**Keywords:** SEM, microCT, desublimation, attenuation, segmentation, soft tissue, histology

## Abstract

Iodine imparts strong contrast to objects imaged with electrons and X-rays due to its high atomic number (53), and is widely used in liquid form as a microscopic stain and clinical contrast agent. We have developed a simple technique which exploits elemental iodine's sublimation-deposition state-change equilibrium to vapor stain specimens with iodine gas. Specimens are enclosed in a gas-tight container along with a small mass of solid I_2_. The bottle is left at ambient laboratory conditions while staining proceeds until empirically determined completion (typically days to weeks). We demonstrate the utility of iodine vapor staining by applying it to resin-embedded tissue blocks and whole locusts and imaging them with backscattered electron scanning electron microscopy (BSE SEM) or X-ray microtomography (XMT). Contrast is comparable to that achieved with liquid staining but without the consequent tissue shrinkage, stain pooling, or uneven coverage artefacts associated with immersing the specimen in iodine solutions. Unmineralized tissue histology can be read in BSE SEM images with good discrimination between tissue components. Organs within the locust head are readily distinguished in XMT images with particularly useful contrast in the chitin exoskeleton, muscle and nerves. Here, we have used iodine vapor staining for two imaging modalities in frequent use in our laboratories and on the specimen types with which we work. It is likely to be equally convenient for a wide range of specimens, and for other modalities which generate contrast from electron- and photon-sample interactions, such as transmission electron microscopy and light microscopy. *Microsc. Res. Tech. 77:1044–1051, 2014*. © 2014 The Authors. Microscopy Research Technique published by Wiley Periodocals, Inc.

## INTRODUCTION

Iodine in one form or another is widely used as a negative or positive contrast agent in electron and X-ray imaging because its high atomic number (53) and mass (126.9 g·mol^−1^) result in strong backscattering of electrons (Boyde and Koole, 2001) and attenuation of X-rays (Hubbell and Seltzer, 1996) compared to the elements which predominate in unmineralized tissues (_1_H, _6_C, _7_N, _8_O). When applied directly on a specimen, iodine distributes according to its affinity for substrate materials which increases mean atomic number (*Z*) in an organelle-, cell-, or tissue-specific manner. To date this has been achieved by applying iodine moieties to a specimen in a liquid form such as alcoholic or aqueous solutions (Boyde, 2012; Jeffery et al., 2011; Lautenschlager et al., 2014; Ley et al., 2014; Metscher, 2009; Pauwels et al., 2013; Richards et al., 2012; Vickerton et al., 2013). However, liquid preparations may cause shrinkage of unembedded tissue (Vickerton et al., 2013), and with embedded samples may limit penetration according to solvent characteristics, or accumulate on surface features, and can only be used on specimens that can tolerate wetting and/or which are able to be submerged.

Elemental iodine (I_2_) is a solid at room temperature and atmospheric pressure, undergoing gradual sublimation into the surrounding atmosphere. Under these conditions, gaseous I_2_ deposits (desublimes) on and reacts with structures in its vicinity. “Fuming” with heated iodine vapor has been used for nearly a century in the forensic recovery of fingerprints and text from paper, which is then observed with visible light (Jasuja and Singh, 2009; Kelly et al., 2012; Mitchell, 1920). We have exploited iodine vapor staining as a simple and inexpensive approach to introduce atomic number contrast to specimens for imaging with backscattered electron scanning electron microscopy (BSE SEM) and X-ray microtomography (XMT). As far as we are aware, this is the first technical description of iodine vapor staining for atomic number contrast.

## METHODS AND RESULTS

### Staining Procedure

Solid elemental iodine (25–250 mg) is placed within a small open glass vial which is included with the sample in a gas-tight container, such as a glass jar with a screw top or a glass-stoppered bottle. We used at QMUL resublimed iodine crystals (BDH Chemicals, Poole, England, product n. 28564) and at RVC resublimed iodine pellets (GPR RECTAPUR®, VWR Leicestershire, UK, cat n. 24755.181). The mass of iodine provided to the system need not be titrated. The container is left sealed at room temperature until the desired staining has occurred, which varies depending on specimen size and type, and imaging modality.

### Scanning Electron Microscopy

*Z* or material contrast in BSE SEM is well known. Increasing *Z* in the spot under the beam increases the BSE signal by causing a greater proportion of the incident electrons to collide elastically within the specimen and reach the BSE detector. Strong BSE SEM contrast is natively produced by the mineral component in calcified tissues but unprocessed and unstained unmineralized tissues generate little BSE signal.

We applied iodine vapor staining to poly-methylmethacrylate (PMMA) embedded blocks of tissue from mouse, rat, horse, and human bone organs to increase *Z* in the unmineralized tissue components. Block surfaces had been prepared for imaging by polishing or diamond ultramilling. Staining was performed in 50–500 mL standard glass jars with screw tops. We imaged the blocks uncoated under high chamber pressure (50 Pa) with a Zeiss EVO MA10 at 20 kV and 0.3–1.0 nA nominal beam current.

Iodine vapor staining results in differential contrast in BSE SEM imaging (Figs. 4).^1^ Muscle and erythrocytes give strong positive contrast, with chondrocyte nuclei and cartilage matrix giving moderate positive contrast. Pericytic and interterritorial regions of cartilage matrix can be distinguished. Annulus fibrosus and nucleus pulposus of the intervertebral disk stain well. Adipocyte cell peripheries can be clearly distinguished. PMMA itself acquires a small increase in backscattering signal, but not so much as to interfere with reading tissue architecture. Iodine staining is limited to the specimen surface. Cracks do not acquire the high signal seen as an artefact with triiodide solution staining (Boyde, 2012; Ley et al., 2014).

**Fig 1:**
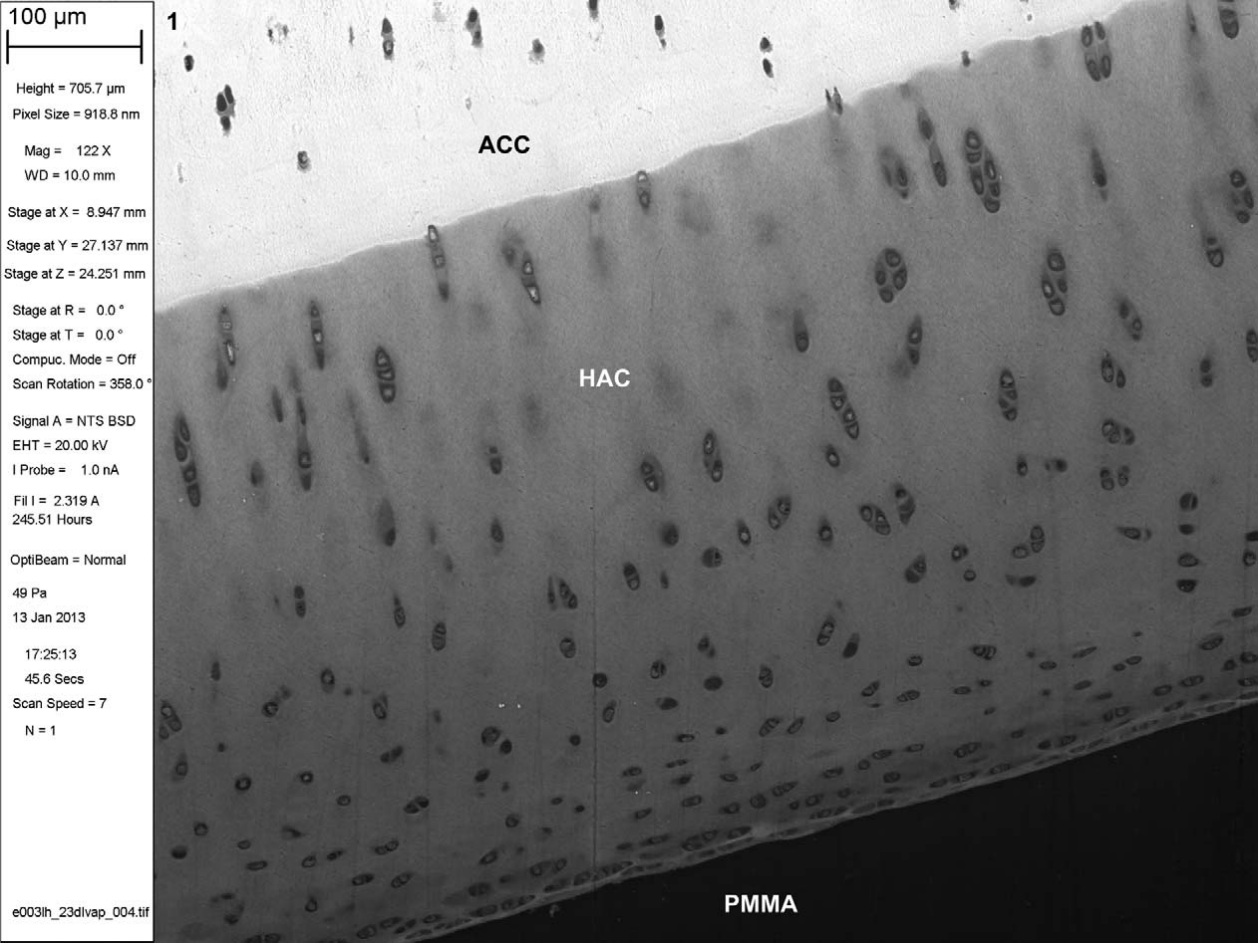
BSE SEM of iodine vapor stained PMMA-embedded musculoskeletal tissues. Three year old Thoroughbred horse distal third metacarpal bone, normal articular cartilage, PMMA block stained with iodine vapor for 23 days. 100 μm scale bar in meta-data panel. ACC = articular calcified cartilage. HAC = hyaline articular cartilage.

**Fig 2:**
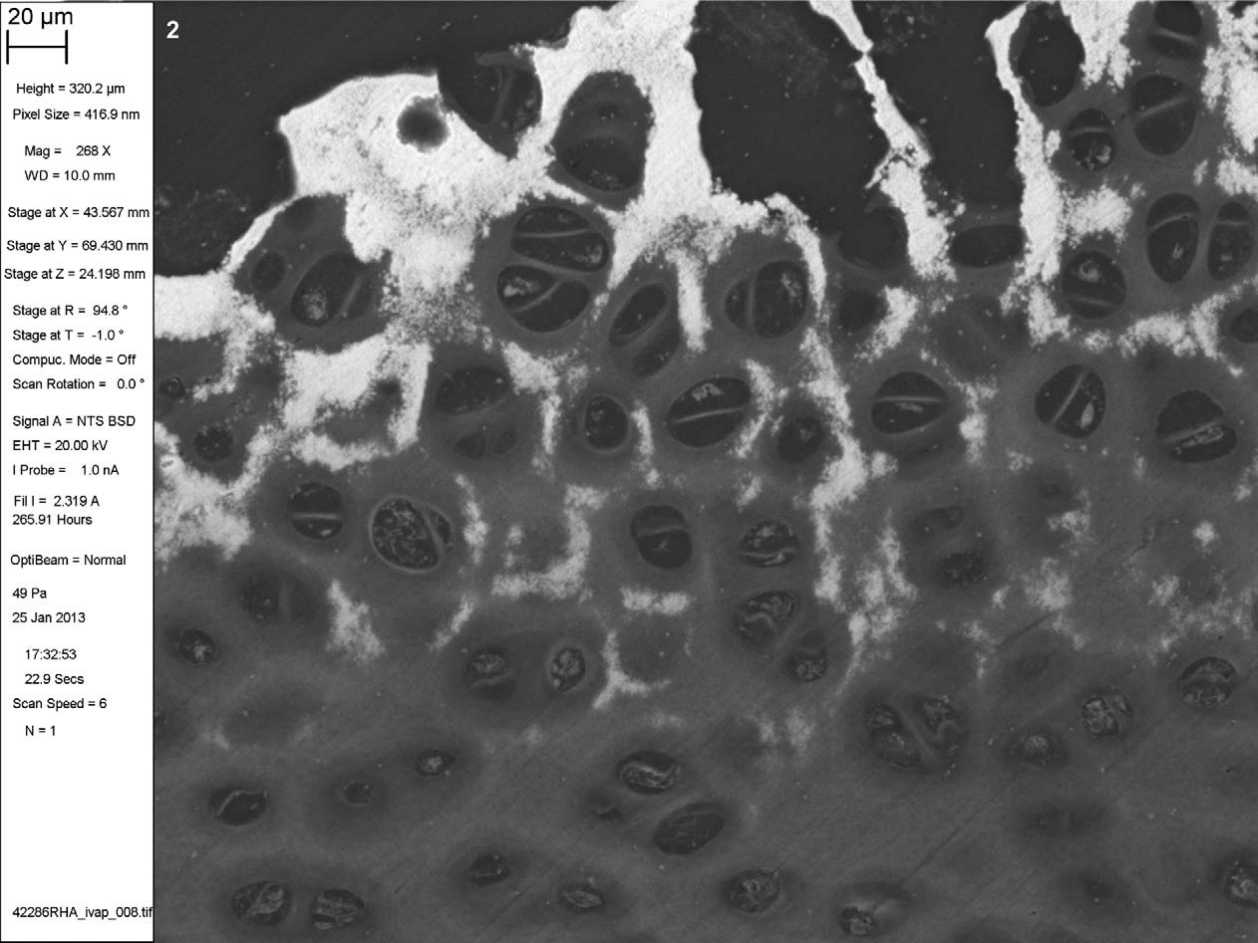
BSE SEM of iodine vapor stained PMMA-embedded musculoskeletal tissues. Neonatal Thoroughbred horse distal third metacarpal bone, mineralizing front of the articular cartilage, PMMA block stained with iodine vapor for 36 days. 20 μm scale bar in meta-data panel.

**Fig 3:**
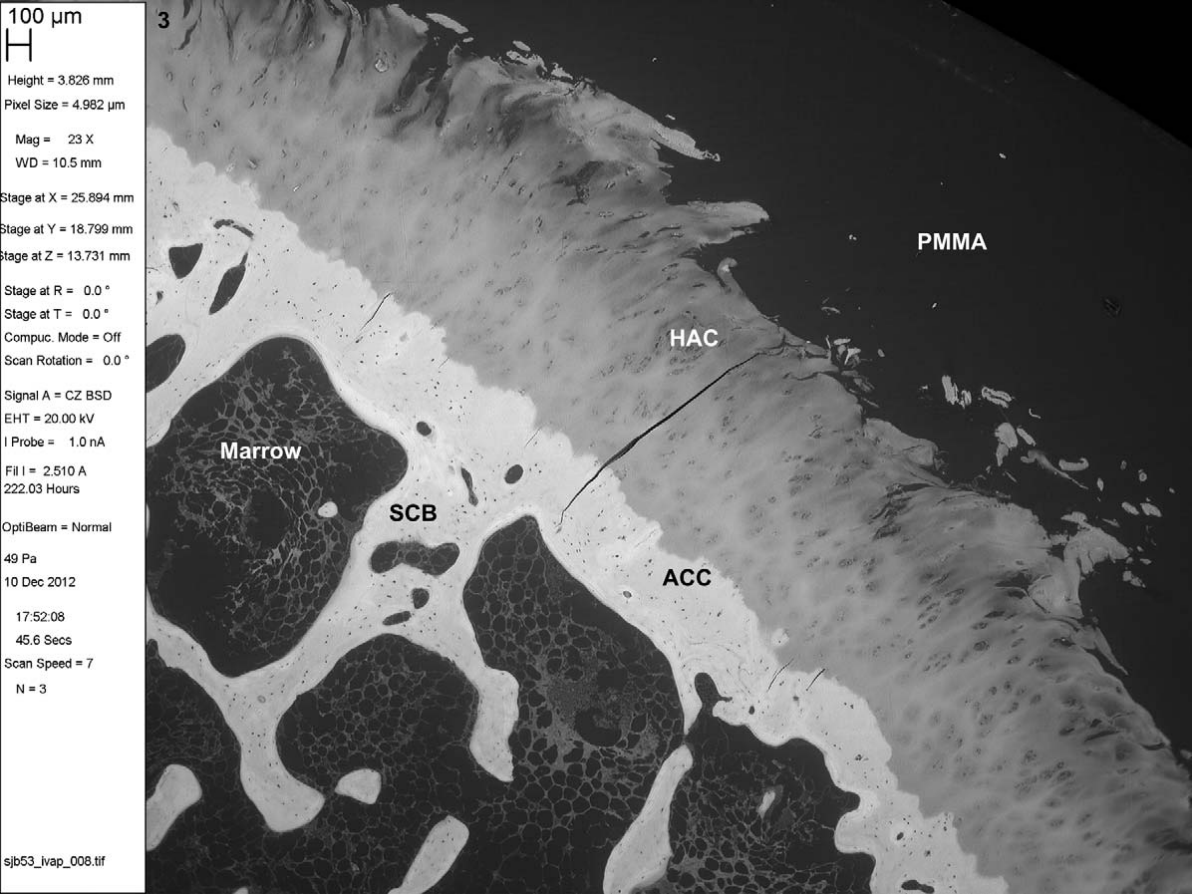
BSE SEM of iodine vapor stained PMMA-embedded musculoskeletal tissues. Adult human femoral head showing typical changes of osteoarthritis with fibrillation of the HAC. PMMA block stained with iodine vapor for 20 days. 100 μm scale bar in meta-data panel. SCB = subchondral bone.

**Fig 4:**
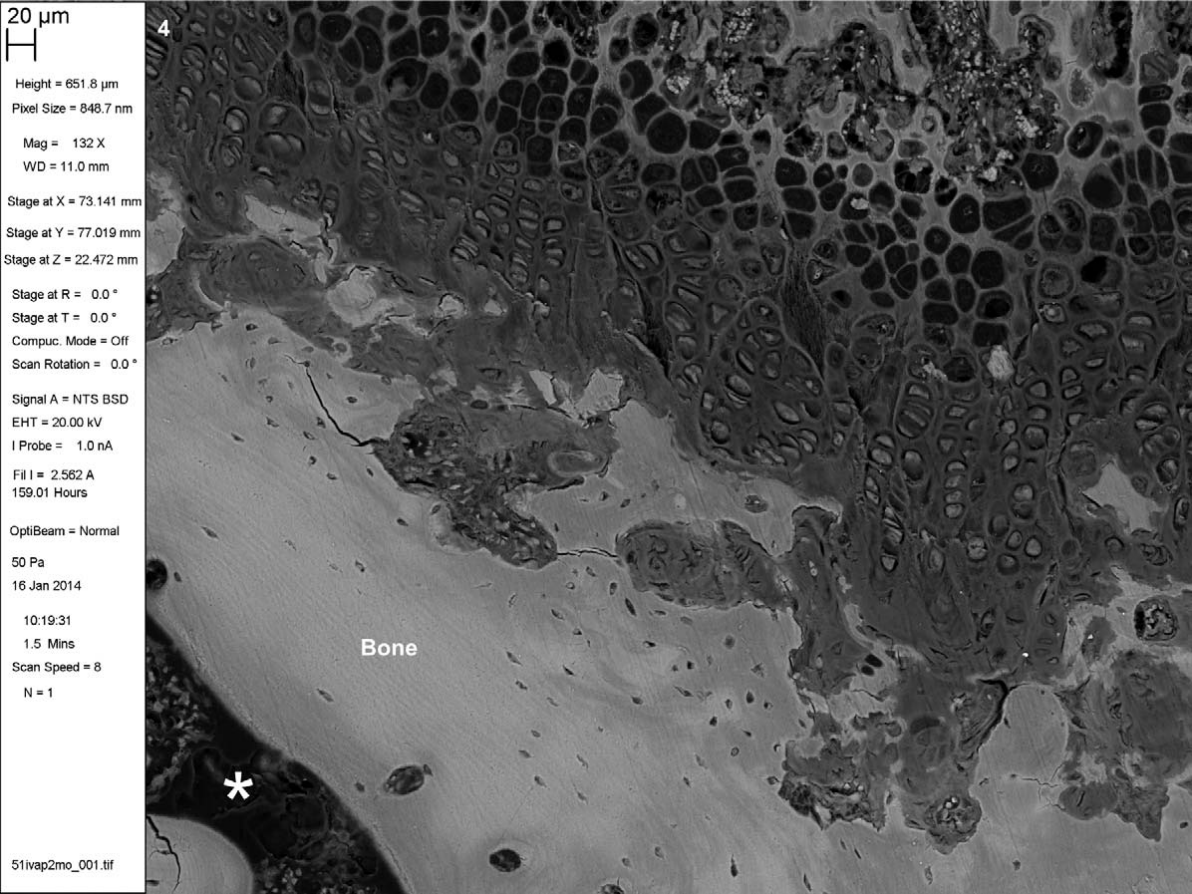
BSE SEM of iodine vapor stained PMMA-embedded musculoskeletal tissues. Rat distal femur growth plate (distal top). 20 μm scale bar in meta-data panel. Asterix ^*^in marrow space in epiphysis. PMMA block stained with iodine vapor for 2 months.

### X-Ray Microtomography

XMT is an imaging technique which nondestructively generates two-dimensional (2D) cross-sectional images (tomograms) from projected radiographs taken at different angles through the specimen (Elliott and Dover, 1982). By stacking a series of tomograms, a 3D image is obtained. XMT relies on differential attenuation or phase shifting of X-ray photons by atoms in the specimen to generate contrast.

For studies on insect flight control, we wished to image the geometry of locust trichoid sensilla which are delicate hairs on the order of 5–10 μm in diameter and 200–400 μm in length found on the frons and vertex of the head (Weis-Fogh, 1949). Wetting locusts in fixative and a nonisotonic solvent by following “wet” protocols risked introducing geometric changes to the trichoids. Additionally, adult locusts are buoyant and resist being submerged in aqueous or ethanolic solutions. To avoid these problems, we applied iodine vapor staining to whole locusts.

Adult locusts of the *Schistocerca* and *Locusta* genera (Blades Biological, Edenbridge, UK) were killed by freezing at −20°C, thawed overnight and placed individually or in pairs on the bottom of a 250 mL wide-mouth glass-stoppered reagent bottle (Schott AG, Mainz, Germany) or a glass-lidded weighing bottle (Lenz Laborglas GmbH & Co. KG, Wertheim, Germany) with 5–10 pellets of elemental iodine, each pellet having a mass of around 25 mg. To prevent gas leaks, we sealed the ground glass joint with a thin layer of PTFE grease (HGT100, Solent Lubricants, Leicester, UK). After incubating at room temperature for 20 h, 7 d, 14 d, or 30 d, locusts were carefully decapitated by dissecting around the neck membrane and through the soft tissues of the neck (Chapman, 1998) with fine scissors. We mounted the head for XMT by melting dental wax around the mouthpieces and the cut stump of the neck, then attaching the wax to a cut-down cryo-tube fit to the rotation stage stub. Using a Skyscan 1172F (Bruker MicroCT, Kontich, Belgium) with its tungsten target X-ray source set to 59 kV and 167 μA, and 0.25 mm Al filter, we positioned the locust head in the beam so as to achieve 1.9–2.5 μm nominal pixel spacing while maintaining the full diameter of the head within the 4000 × 2672 detector element camera frame for the rotation. The head was rotated through 180° or 360° at 0.10° – 0.15° increments with 1–4 × averaging and 3.5–14 s total exposure per projection. Tomographic reconstructions were calculated with NRecon (SkyScan, v1.6.9) on the GPU (NVIDIA GTX 680, EVGA) with default settings and visualized as tomographic slices in ImageJ v1.48p (Schneider et al., 2012) and 3D renderings in Drishti v2.4 (Limaye, 2012).

**Fig 5:**
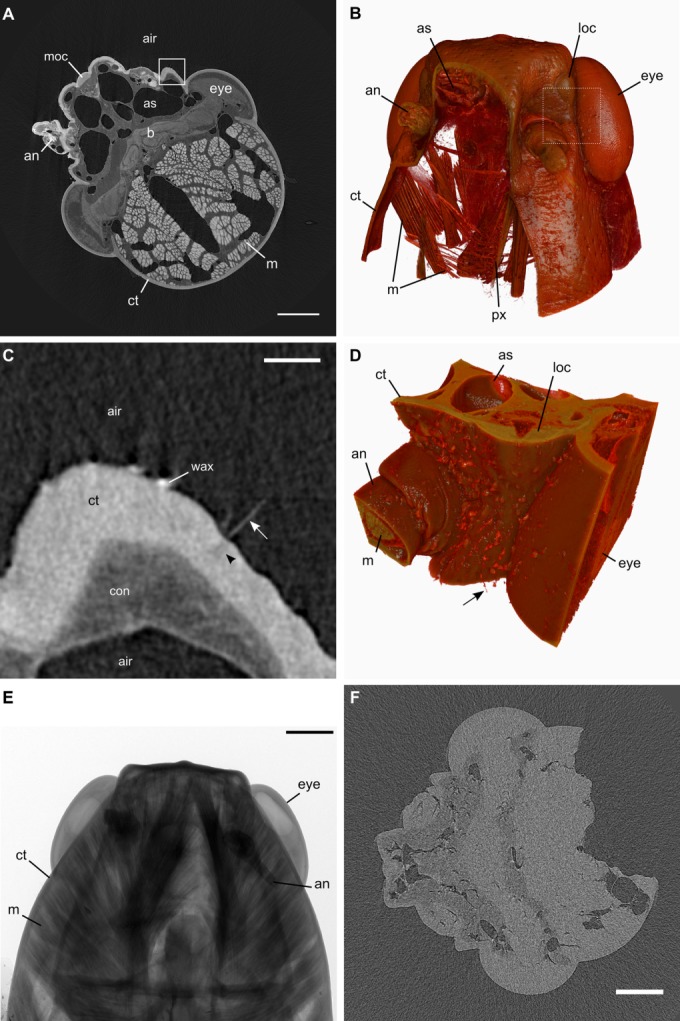
X-ray microtomography of locust head. A: tomogram of *Locusta* head exposed to iodine vapor for 7d. Note contrast between tissue and air and among tissue types. Box shown magnified in (C). B: 3D rendering of 8× downsampled tomograms from the series shown in (A), in frontal view with a cutaway through the right frons and antenna showing the internal muscular structures of the pharynx. Box shown magnified in (D). C: Detail from box in (A) showing trichoid sensillum as it curves through the tomographic plane (arrow) and the space in the cuticle through which its sensory dendrite passes (arrowhead). D: Detail from box in (B) without downsampling of original tomogram showing trichoid sensilla (arrow) on external surface and internal anatomy. E: Example projection image from which tomograms were calculated. Note attenuation of X-ray beam by stained structures F: Tomogram of unstained *Schistocerca* head at similar anatomic plane to (A) displaying contrast between tissue and air but little detail within soft tissue. Noise is due to a faster acquisition (1600 ms per projection) and larger angular increment (0.6°) than in (A). Key: ct, cuticle; m, muscle; b, brain; con, connective tissue; as, air sac; an, antenna; loc, left ocellus; moc, median ocellus; eye, compound eye; px, pharynx. Scale bars 1 mm (A, E, F) and 100 μm (C). [Color figure can be viewed in the online issue, which is available at wileyonlinelibrary.com.]

Iodine vapor staining imparted strong positive contrast to insect cuticle and muscle, moderate positive contrast to neural tissue of the eye, nerves, ganglia, and brain, and allowed segmentation and identification of fine tissue structures (Fig. 5). No practical difference in staining efficacy was noted after 7 d; however, an overnight exposure of 20h was insufficient to generate adequate contrast for XMT.

## DISCUSSION

Iodine vapor staining exploits iodine's sublimation-deposition state-change equilibrium at ambient laboratory conditions and is simple and cheap to perform. As little as a single 25 mg pellet of solid elemental I_2_ is required per specimen and the price of 100 g is £15–£50, which might well outlast the lifetime of the laboratory or its occupants. Glass jars are very cheap. Glass-stopper bottles are on the order of £5–£50 and can be reused nearly indefinitely. No other special equipment is required. No solutions need to be prepared and the specimens, depending on stability, do not need to be fixed or permeabilized. Staining intensity can be controlled by the duration of exposure to iodine gas, and sequential imaging may be performed with additional iodine exposure between imaging sessions. Furthermore, solid I_2_ may be added to the chamber to increase the duration of supply so that adequate staining can occur. Staining progress can be monitored qualitatively by observation of an orange or brown color change in the specimen over the course of days to weeks, and eventually a dark grey color after several weeks. Vapor staining is a dry protocol which avoids problems related to wetting specimens with liquids.

Iodine remains labile after depositing on to the specimen and can sublime out of it, which we have observed as staining of unused plastic containers in which stained specimens have been stored, and the gradual reduction of brown coloration of specimens. All iodine-induced color was lost from vapor-stained PMMA blocks stored for 2 years. I_2_ is extremely mobile in plastic media, and can escape from and through polymer containers (particularly through polyethylene), so we prefer glass containers to prevent accidental staining of walls and shelves by escaped I_2_. Organic sealant material such as Parafilm (Pechiney Plastic Packaging Company, Chicago, IL) does not prevent iodine from escaping, because I_2_ diffuses through it, staining it a pink color. Ideally, extraneous plastic ware should be omitted from the protocol so that only the specimen and glass are in contact with solid or gaseous I_2_. However, iodine staining of polymer materials could be used to advantage for imaging plastic parts in engineering applications.

The technique is insensitive to the mass of I_2_ added to the vessel because the partial pressure of iodine vapor at a given temperature is independent of the amount of solid iodine present (Masterton and Hurley, 2008). The only requirement is to provide an adequate mass of solid I_2_ to reach the equilibrium constant for sublimation, and enough to impart staining to the specimen. The specimen acts as a “sink” for gaseous I_2_, which is replaced by further sublimation from solid I_2_ to reestablish equilibrium by Le Châtelier's principle. Staining evenness and rate might be enhanced by modifying solid iodine surface area, staining temperature, chamber volume relative to specimen size, or by optimizing the flow of gas from the solid iodine to the specimen by an arrangement of racks, chambers, or channels. The difference in staining depth between locusts, which were stained evenly through their volume, and PMMA blocks, which were stained only on their surface, may relate to iodine's relatively slower diffusion through thick, solid PMMA compared to hydrated locusts, the latter of which also has a tracheal network delivering gas to body tissues.

The explanation for iodine's affinity for different substrates is likely to be a combination of factors, including its variable solubility in polar and nonpolar solvents, its tendency to be reduced from I_2_ to iodide (I^−^) and triiodide (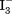) ions (and hence form ionic bonds with specimen cations), its well-known ability to form complexes with starch and glycogen (Jeffery et al., 2011; Lecker et al., 1997), the formation of organoiodine compounds (after I_2_ reacts with C―C double bonds in unsaturated molecules), and its ability to displace covalently bound chlorine atoms.

Dimensional change due to iodine staining cannot occur in PMMA embedded tissue, beyond any shrinkage which has already occurred during dehydration, solvent substitution, and polymerization. It is well established that freeze drying causes the least volumetric change during sample preparation and we propose that a good approach would be to use iodine vapor staining of freeze-dried tissue blocks. In this study, we did not observe the dramatic shrinkage which can occur when submerging mammalian soft tissue specimens in iodine solutions (Vickerton et al., 2013). However, we studied insect exoskeletal elements and further quantitative studies would be needed to confirm that this advantage would apply to, for example, previously freeze-dried tissue samples, although these will have shrunken by approximately 20% in volume during the freeze-drying process (Boyde and Franc, 1981).

### Backscattered Electron SEM

Iodine vapor staining for BSE SEM is well suited to tissue blocks which have been previously embedded in a polymer such as PMMA, glycol methacrylate, or epoxy resin. We have very extensive experience with PMMA-embedded skeletal and dental calcified tissues where the original intent was to study variations in mineral concentration using quantitative backscattered electron (qBSE) SEM. Without additional staining, we normally only visualize the calcium phosphate mineral component within the hard tissue matrix. After vapor phase staining, we can read all soft tissue histological features such as cells and unmineralized organic matrix, as, for example, osteoid in bone, and hyaline cartilage in joints and growth plates. Vapor staining has advantages over “wet” staining with iodine solutions which tend to pool around cracks and defects in the sample surface causing a high BSE signal artefact. However, the spatial extent of vapor staining cannot be as readily controlled as liquid staining, which may be confined to a single droplet placed on the specimen surface.

Iodine contrast has been used in SEM by its being incorporated into a methacrylate resin (Boyde and Koole, 2001). In this manner, it can give contrast to embedded material and/or form a strongly backscattering cast after the biological tissue has been macerated. Halogenated dimethacrylate resins (Davy, 1994) have been used extensively as *Z*-standards for qBSE SEM studies of mineralized tissues, with the iodinated dimethacrylate resin forming a convenient upper bound for bone and dentine (Howell et al., 1998).

### X-Ray Microtomography

**Fig 6:**
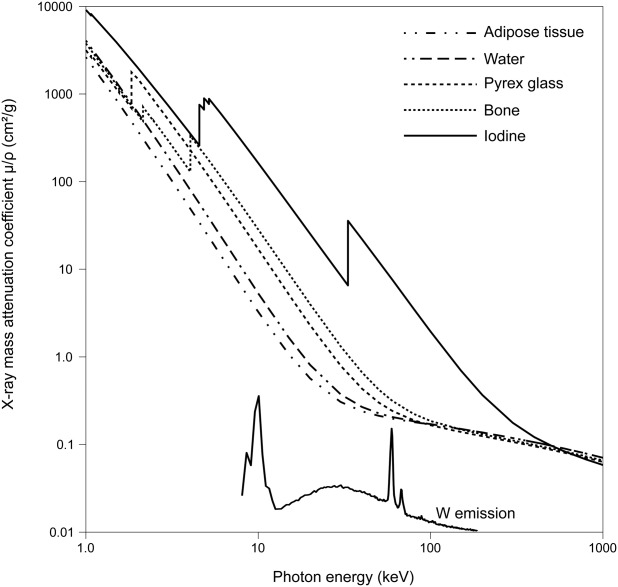
X-ray attenuation and emission spectra. X-ray mass attenuation coefficients of adipose tissue, water, Pyrex glass, bone, and elemental iodine show how iodine has a 5- to 30-fold stronger attenuation than biological materials and glass in the range of photon energies produced by a tungsten target (W emission). Figure generated de novo from data published in Hammersberg et al., Linköping Electron Artic Mech Eng, 1998, 1, 1–13, and Hubbell and Seltzer, 1996.

Iodine vapor staining is highly suited to XMT studies of whole insects. The L1- (5.2 keV) and K-edges (33.2 keV) of iodine's mass attenuation coefficient match well the typical photon energies generated by laboratory and clinical X-ray sources, and in particular the Bremsstrahlung and K lines (58.0 and 59.3 keV) of tungsten's emission spectrum (Fig. 6; Hammersberg et al., 1998; Hubbell and Seltzer, 1996; Lusic and Grinstaff, 2013; Williams, 2009). Previous experiments on insect pupae and adults used iodine solutions (Lugol's iodine / IKI: 1% I_2_ and 2% KI in water) or tinctures (1% I_2_ in 70% or 100% ethanol, or 100% methanol) in which animals were immersed for up to 7 days. The resulting tomograms allowed for detailed “virtual dissection” of the animals because iodine differentially localized within anatomical structures (Metscher, 2009; Richards et al., 2012). Vapor staining gives comparable contrast to liquid staining with muscle and cuticle giving particularly strong positive contrast. As noted by Jeffery et al. (2011), individual muscle fascicles can be distinguished and differentiation between fatty and watery parts of the brain can be made. Nerves and ganglia can be traced through less-attenuating surrounding tissue.

Penetration is even and effective by 7 d, which is comparable with the 7 d treatment used by Richards et al. (2012) but slower than the overnight staining reported by Metscher (2009) and the 24 h staining used by Pauwels et al. (2013). In our initial trials, we attempted to enhance iodine deposition on the cuticle surface by preheating the sealed bottle and solid iodine to 50°C and placing a frozen (−20°C) locust into it, then leaving it at room temperature for 20 h (overnight); however, staining was weak and so we instead settled on the simpler room-temperature protocol. We did not fix the locusts, yet they appeared to maintain their morphology after 1 month of staining, perhaps due to the animals' own resistance to desiccation and to the antimicrobial properties of I_2_ preventing bacterial and fungal decay and its denaturing of proteins slowing autolysis (Cooper, 2007).

Current alternatives to iodine for X-ray contrast in biological soft tissues include phase contrast (Marenzana et al., 2014), phosphotungstic acid (Metscher, 2009), and a number of newly investigated compounds such as mercury chloride, phosphomolybdic acid, and ammonium orthomolybdate (Pauwels et al., 2013). All other chemical agents require submersion of the specimen in aqueous or alcoholic solution.

Movement artefact during long XMT exposures was the major drawback to this method of imaging, which might relate to the relatively low flux source (10 W maximum) and high spatial resolution required to image trichoids leading to 17–18 h scans, and consequent high likelihood of small movements of the specimen. Sources of movement artefact were tissue shrinkage due to dehydration through the cut stump of the neck and the head shifting in its wax mount. We attempted to ameliorate these causes by “soldering” the cut stump at the base of the head into a waxy cradle and melting the wax before inserting the mouthpieces. Our exposure parameters were similar to those previously reported (Metscher, 2009; Richards et al., 2012).

## Footnotes

Iodine vapor staining imparts differential contrast into tissue and cell components allowing reading of soft tissue histology in BSE SEM. Here, we demonstrate use of the technique on undemineralized tissues where iodine vapor staining has allowed direct visualization of soft tissues in the context of mineralized compartments. Without iodine vapor staining, only mineralized tissues such as bone and articular calcified cartilage generate sufficient BSE signal to be detected as lighter shades against poorly backscattering PMMA and soft tissues.
